# Effect of Porosity and Concentration Polarization on Electrolyte Diffusive Transport Parameters through Ceramic Membranes with Similar Nanopore Size

**DOI:** 10.3390/nano4030700

**Published:** 2014-08-06

**Authors:** Virginia Romero, Victor Vega, Javier García, Victor M. Prida, Blanca Hernando, Juana Benavente

**Affiliations:** 1 Applied Physics Department I, Faculty of Sciences, University of Malaga, E-29071 Málaga, Spain; E-Mail: virgirom@uma.es; 2 Physics Department, Faculty of Sciences, University of Oviedo, E-33007 Oviedo, Spain; E-Mails: vegavictor@uniovi.es (V.V.); garciaferjavier.uo@uniovi.es (J.G.); vmpp@uniovi.es (V.M.P.); grande@uniovi.es (B.H.)

**Keywords:** nanoporous alumina membranes (NPAMs), atomic layer deposition (ALD) surface coating, membrane potentials, concentration polarization

## Abstract

Diffusive transport through nanoporous alumina membranes (NPAMs) produced by the two-step anodization method, with similar pore size but different porosity, is studied by analyzing membrane potential measured with NaCl solutions at different concentrations. Donnan exclusion of co-ions at the solution/membrane interface seem to exert a certain control on the diffusive transport of ions through NPAMs with low porosity, which might be reduced by coating the membrane surface with appropriated materials, as it is the case of SiO_2_. Our results also show the effect of concentration polarization at the membrane surface on ionic transport numbers (or diffusion coefficients) for low-porosity and high electrolyte affinity membranes, which could mask values of those characteristic electrochemical parameters.

## 1. Introduction

Nanoporous alumina membranes (NPAMs), which are obtained through the two-step electrochemical anodization method of aluminum foils, exhibit highly ordered pore arrays with a honeycomb structure, having pores radii usually in the range from 10 nm to 100 nm and thickness between 10 μm and 100 μm [1,2]. The well-defined porous structure of NPAMs and the possibility of modification of both pore size and surface nature by atomic layer deposition (ALD) technique [3,4] have lately increased their application in molecular release and sensor devices in liquid media [5,6,7]. In these applications, NPAMs are in contact with solutions containing molecules (both neutral and charged, and even ions), which flow along the membrane pores due to concentration gradients. However, concentration-polarization, that is, the formation of a solution stagnant layer with a concentration profile near the membrane surface, might affect more or less significantly the concentration gradient depending on the real value of concentration at the membrane surface, *C*_f_^m^, with respect to the bulk feed concentration, *C*_f_, which is the parameter that can be experimentally adjusted. Consequently, the solute transport may also be affected, mainly in the case of low porosity membranes and charged species, due to electrical interactions, which could mask the true values of the evaluated transport parameters.

In this study, we analyze the effect of membrane porosity in the diffusive transport of ions through three NPAMs having different porosity but with similar pore radii (~10 nm) and thickness (~60 μm), as well as the effect of surface functionalization/modification (SiO_2_ coverage by ALD) for a sample with final pore size similar to the alumina ones. Transport characterization was carried out by analyzing membrane potential measurements performed with NaCl solutions at different concentrations, which allow the evaluation of diffusive parameters (ions transport numbers or diffusion coefficients) [8]. Moreover, interfacial effects have also been considered by comparing membrane potential values obtained with stirred and non-stirred solutions. Differences in diffusive parameters determined for the different samples could provide information about the influence of interfacial effects on the diffusive transport of charged species. These results allow for a better comprehension of the diffusive ionic transport across nanoporous membranes, which is of great importance for mass transport processes associated to concentration gradients in microfluidics and drug delivery applications.

## 2. Results and Discussion

### 2.1. Microstructure and Morphological Parameters of Alumina Membranes

Three NPAMs fabricated by the anodization process have been studied: one of them is a commercial sample (Anopore™ by Whatman International Ltd., Maidstone, UK), and the two other were synthesized in our laboratory by two-step anodization either in sulfuric (Al-Sf) or oxalic (Al-Ox) acids. Samples of the Al-Ox membranes were also coated with a thin layer of 5 nm in thickness of Al_2_O_3_ or SiO_2_, both deposited by ALD, and the resulting samples will be hereafter referred to as Al-Ox/Al_2_O_3_ and Al-Ox/SiO_2_, respectively. Contact angle measurements revealed substantial differences between SiO_2_ coated membranes and uncoated ones, evidencing the hydrophobic character or silica [8]. Geometrical parameters of the Anopore membrane given by supplier are: pore radii of 10 nm, thickness of 60 μm and porosity between 25% and 50%, although an estimated porosity of 30% seems to be a more accurate value according to indications given by Bluhm *et al.* [9] and from tritiated water diffusion results [10].

**Figure 1:**
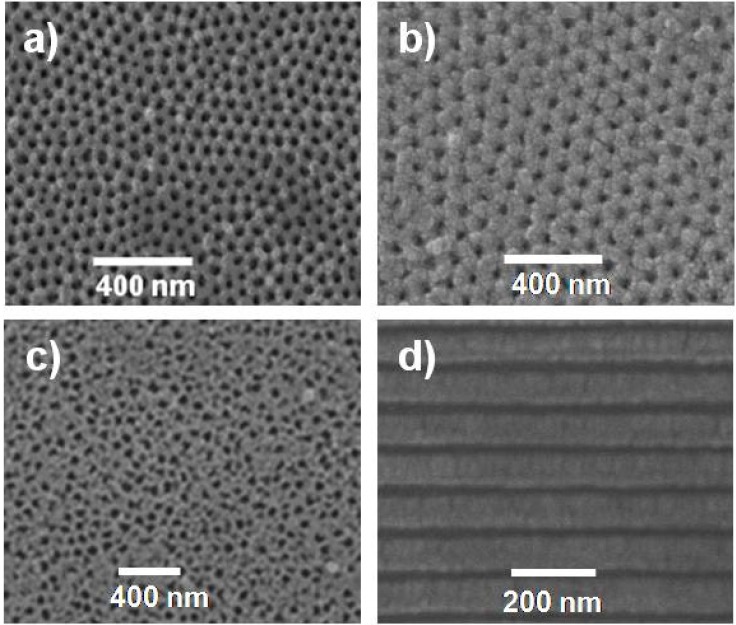
Scanning electron microscopy (SEM) top view images of nanoporous alumina membranes (NPAMs): (**a**) Al-Sf; (**b**) Al-Ox/SiO_2_; (**c**) Anopore; and (**d**) cross-sectional SEM image of sample Al-Ox/SiO_2_.

Figure 1 shows the top-view scanning electron microscopy (SEM) images of Al-Sf and Al-Ox/SiO_2_ samples, as well as for the commercial Anopore membrane. As it can be observed, both experimental samples exhibit a well-defined and regular porous structure, rather different to that shown by the commercial membrane (Anopore). Figure 1d displays a cross-sectional SEM image of sample Al-Ox/SiO_2_ evidencing the cylindrical and straight pore channels of the experimental membranes.

Morphological surface parameters for the different samples (pore radii, *r*_p_, and interpore distance, *D*_int_) as well as membrane thickness (∆*x*) were determined from SEM micrographs analysis and the values obtained are collected in Table 1. Membrane porosity (%) was determined by using the following expression [11]: 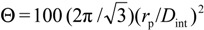. The estimated average porosity values (<Θ>) have been obtained by considering the porosity from both, top and bottom, SEM surface images.

**Table 1:** Morphological parameters characteristic of the studied NPAMs: pore radius (*r*_p_ = *d*_p_/2), interpore distance (*D*_int_), thickness (∆*x*) and estimated average porosity (<Θ>).

Sample	*r*_p_ (nm)	*D*_int_ (nm)	(<Θ>) (%)
Al-Sf	12 ± 2	65 ± 2	15
Al-Ox + Al_2_O_3_	11 ± 3	105 ± 3	5
Al-Ox + SiO_2_	11 ± 3	105 ± 3	5

### 2.2. Characterization of Diffusive Transport across the Nanoporous Membranes

Fixed charge on both external surfaces and pore wall (or internal surface) as well as membrane structure can significantly influence the transport of electrolyte solutions and/or charged species across membranes [12,13]. Effective fixed charge, *X*_ef_, and ion transport number, *t_i_*, or fraction of the total current transported for one ion (*t_i_* = *I_i_*/*I*_T_*)* are two significant parameters that can be determined from membrane potential values (∆Ф_mbr_).

According to the Teorell-Meyer-Sievers (TMS) theory [14,15], membrane potential can be considered as the sum of two Donnan potentials (one at each membrane-solution interface), associated to the exclusion of the co-ions (or ions of the same sign as the membrane charge), plus a diffusion potential in the membrane due to the different mobility of the ions inside the membrane pores, that is: ∆Ф_mbr_ = ∆ø_Don(I)_ + ∆ø_dif_ + ∆ø_Don(II)_. In the following expressions, 1:1 electrolytes (|*z*_+_| = |*z*_−_| = 1) and diluted solutions (herein, concentrations are used instead of activities) will be considered.

- The Donnan potential for a positively charged membrane with effective fixed charge *X*_ef_ in contact with an electrolyte solution of concentration *C* can be expressed as [16]:
∆ø_Don(I)_ = (R*T*/F)ln[*C*^m^ / *C*] = (R*T* /F)ln[(*X*_ef_ / 2*C*) + [(*X*_ef_ / 2*C*)^2^ + 1]^1/2^] (1)
  where R and F correspond to the gas and Faraday constants, and *T* is the temperature of the system, while *C*^m^ represents the concentration in the membrane, related with *X*_ef_ and *C* by the electroneutrality condition [16]: *X*_ef_ + *z*_+_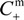 = *z*_−_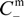.
- The diffusion potential is given by [16]:
∆ø_dif_ = (R*T*/F)ln[(*t*_−_ − *t*_+_)]ln(*C*_c_/*C*_v_) = (R*T*/F)[(2*t* − 1)]ln(*C*_c_/*C*_v_) (2)
  where *t*_+_ and *t*_−_ are the cation and anion transport numbers in the membrane, respectively. According to transport number definition, *t*_+_ + *t*_−_ = 1, and for single salts: *t*_−_ = 1 − *t*_+_.

Taking into account Equations (1) and (2), the membrane potential can be expressed as [16]:

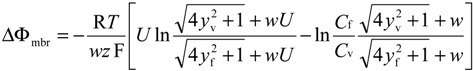
(3)
where *w* = +1/−1 for positively/negatively charged membranes, *y_j_* = *C_j_*/*X*_ef_ and the parameter *U* is related to the ions transport numbers (*t_i_*) and diffusion coefficients (*D_i_*) by the following expression: *U* = *t*_+_ − *t*_−_ = 2*t*_+_ − 1 = (*D*_+_ − *D*_−_)/(*D*_+_ + *D*_−_), for 1:1 electrolytes.

**Figure 2:**
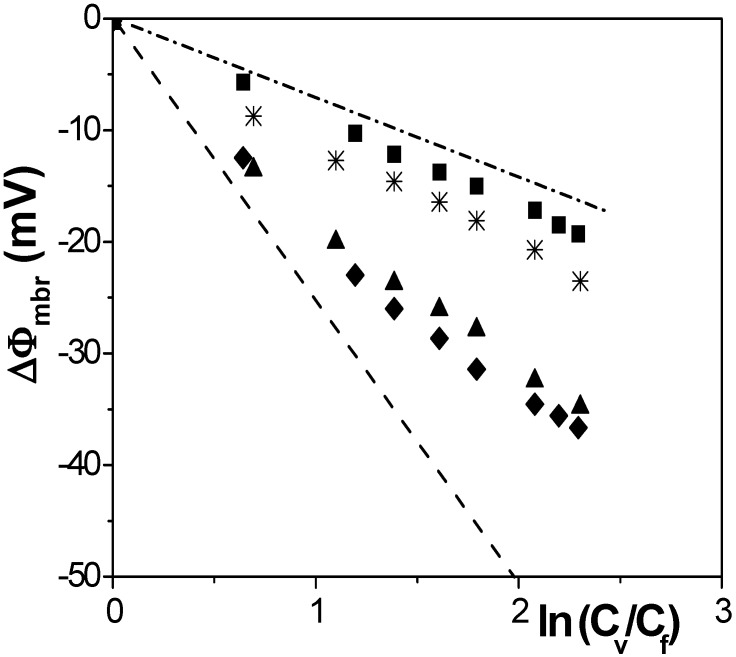
Membrane potential as a function of solution concentrations ratio for different membranes: Anopore (■), Al-Sf (♦), Al-Ox/Al_2_O_3_ (▲), and Al-Ox/SiO_2_ (*).

Figure 2 shows membrane potentials as a function of the concentration ratio for the studied membranes. For comparison, membrane potential for an ideal anion-exchanger membrane (dashed line) and the solution diffusion potentials (dashed-dot line) are also represented in Figure 2. These parameter values were determined by using in Equation (1) the following values: *t*_−_ = 1 for ideal anion-exchanger, and the solution transport number *t*_+_ = 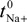 [17] in the case of solution diffusion potentials. As it can be observed, significant differences in ∆Ф_mbr_ values were obtained depending on both membrane porosity and surface nature (consequently, different ions-membrane electroaffinity). Particularly, very similar membrane potentials have been obtained for Al-Sf and Al-Ox/Al_2_O_3_ samples, that is, for nanoporous membranes with alumina surfaces, similar pore radii and low porosity (15% and 5%, respectively); however, much lower ∆Ф_mbr_ values for the same concentration ratio were obtained for the alumina membrane with higher porosity (30%), and they are very similar to the solution diffusion potential, which is an indication of the small barrier effect of the Anopore membrane to the transport of ions. On the other hand, similar values were also obtained for the SiO_2_ coated surface sample (Al-Ox/SiO_2_ membrane), with significantly lower porosity (5%), and in this case they might be associated to a reduction in the electrolyte/membrane electrical interactions as a result of the SiO_2_ coating.

**Figure 3:**
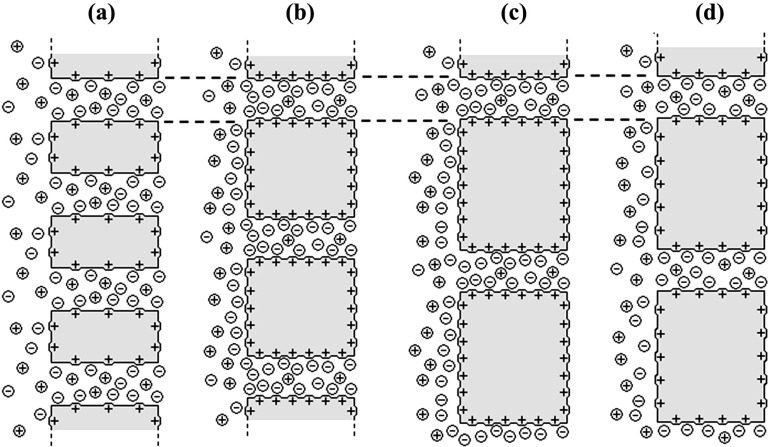
Illustrative representation of diffusive ion transport through membranes with different porosities and positive surface fixed charge: (**a**) Anopore (high porosity and low co-ion exclusion); (**b**) Al-Sf (medium porosity and high co-ion exclusion); (**c**) Al-Ox/Al_2_O_3_ (low porosity and high co-ion exclusion); and (**d**) Al-Ox/SiO_2_ (low porosity and low co-ion exclusion).

Differences in the diffusive ionic transport across the studied membranes are attributed to membranes structure and their surface electrical nature. Although all membranes have similar pore radii, their different interpore distance or porosity as well as surface charge might affect the co-ion exclusion from the interface and the pores, which would increase the counter-ion presence in both interface and pore solutions, as depicted in Figure 3, which schematically shows the ionic transport behavior of the different membranes.

The fit of the experimental values shown in Figure 2, by using Equation (3), allows us the estimation of effective fixed charge, *X*_ef_, and anion transport number, *t*_−_, values for each membrane, which are indicated in Table 2. For all membranes, *t*_−_ values are higher than the solution average value 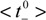
= 0.615 ± 0.004 [17]), which is an indication of the electropositive character of all the samples. Taking into account the relationship between ion transport numbers and diffusion coefficients [17]: *t_i_* = *D_i_*/(*D*_+_ + *D*_−_), ion diffusion ratio for each membrane (*D*_−_/*D*_+_ = *t*_−_/*t*_+_) was also estimated and their values are also indicated in Table 2. It should be pointed out that the value of cation diffusion coefficient through the Al-Sf membrane hardly differs from that previously reported for this sample and determined from radiotracer (^22^Na^+^) diffusion measurement (*D*_Na+_^Al-sf^ = 2.8 × 10^−10^ m^2^/s, [18]), which confirms the reliability of the obtained results.

**Table 2:** Effective fixed charge (*X*_ef_), anion transport number (*t*_−_), ionic diffusion coefficients ratio (*D*_−_/*D*_+_) and ions diffusion coefficient values (*D*_−_ and *D*_+_).

Sample	*X*_ef_ (M)	*t*_−_	*D*_−_/*D*_+_	*D*_−_ (m^2^/s)	*D*_+_ (m^2^/s)
Anopore	0.001	0.655	1.90	1.9 × 10^−9^	1.0 × 10^−9^
Al-Sf	0.012	0.751	3.02	9.8 × 10^−^^10^	3.3 × 10^−^^10^
Al-Ox/Al_2_O_3_	0.012	0.724	2.66	9.0 × 10^−^^10^	3.4 × 10^−10^
Al-Ox + SiO_2_	0.003	0.668	2.01	1.4 × 10^−9^	7.0 × 10^−10^

**Figure 4:**
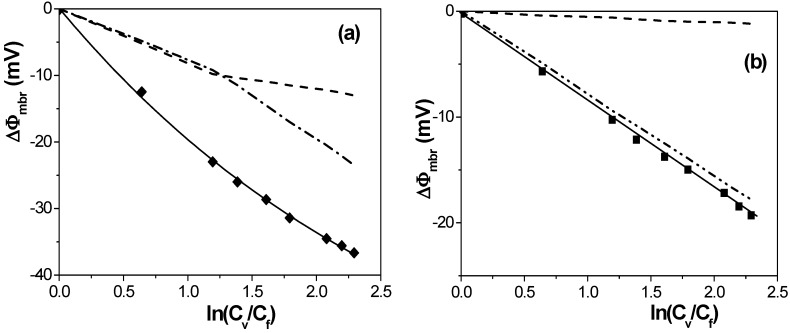
Experimental (symbols) and fitted (solid lines) membrane potentials as a function of solution concentration ratio, plus calculated values for Donnan (dashed lines) and diffusion (dashed-dot lines) contributions determined using Equations (1) and (2) and parameters in Table 2: (**a**) Al-Sf membrane; and (**b**) Anopore membrane.

Differences between interfacial (Donnan) and transport contributions to the total membrane potential depending on the membrane structure can be observed in Figure 4. This figure presents a comparison between experimental and fitted values of the membrane potential, as well as the individual contribution of Donnan and diffusion potential (dashed and dot-dashed lines, respectively), calculated by using Equations (1) and (2) with the corresponding fitted parameters for Al-Sf and Anopore membranes. As it can be observed, for the Al-Sf sample both Donnan and diffusion potentials present practically similar contribution for *C*_v_ ≤ 0.04 M, but the diffusion potential increases more significantly by increasing the concentration gradient. However, for the low charged Anopore sample, the interfacial effect associated to Donnan potential hardly contributes to the membrane potential, which almost coincides with the diffusion potential contribution.

**Figure 5:**
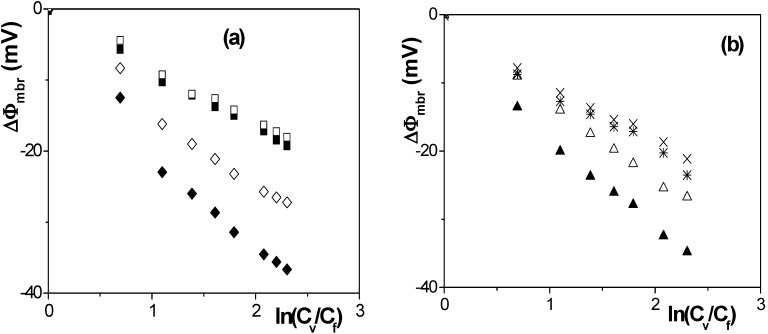
Membrane potential as a function of solution concentrations ratio measured with: (**a**) stirred solutions, Anopore (■), Al-Sf (♦), and non-stirred solutions, Anopore (□), Al-Sf (◊); and (**b**) stirred solutions, Al-Ox/Al_2_O_3_ (▲), Al-Ox/SiO_2_ (*), and non-stirred solutions, Al-Ox/Al_2_O_3_ (∆), Al-Ox/SiO_2_ (×).

Differences in the barrier behavior of the studied membranes can also be observed in Figure 5, where a comparison between membrane potentials measured with stirred and non-stirred solutions is also presented.

**Figure 6:**
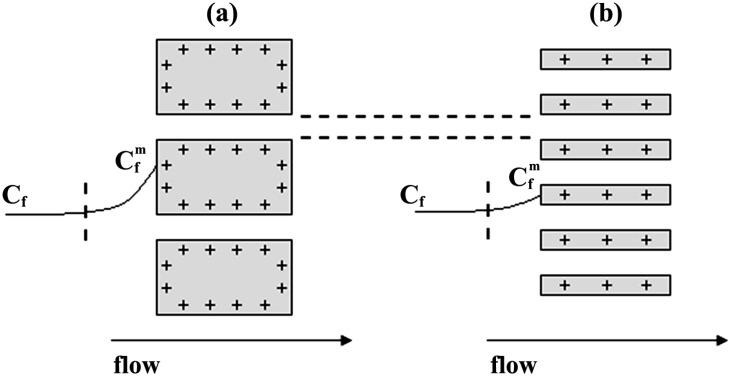
Schematic representation of solution concentration profiles near membranes with similar pore size and: (**a**) high fixed charge and low porosity; and (**b**) low fixed charge and high porosity.

According to the results shown in Figure 5, concentration-polarization (or the concentration profile in the feed solution near the membrane surface) seems to affect the membrane potential values for Al-Sf and Al-Ox/Al_2_O_3_ samples. It is due to their higher effective charge and/or lower porosity by modifying the concentration at the membrane surface with respect to bulk solution, but it hardly affects to the values determined for ANP and Al-Ox/SiO_2_ membranes, as it is schematically indicated in Figure 6 for membranes with similar pore radii. Concentration-polarization, which is a common effect in all membrane separation processes due to the different transport characteristics of solutions (fluids in general) and membrane phases [19], would affect to the ∆Ф_mbr_ values by considering non-correct concentration values as well as by increasing the screening effect on membranes fixed charge.

**Figure 7:**
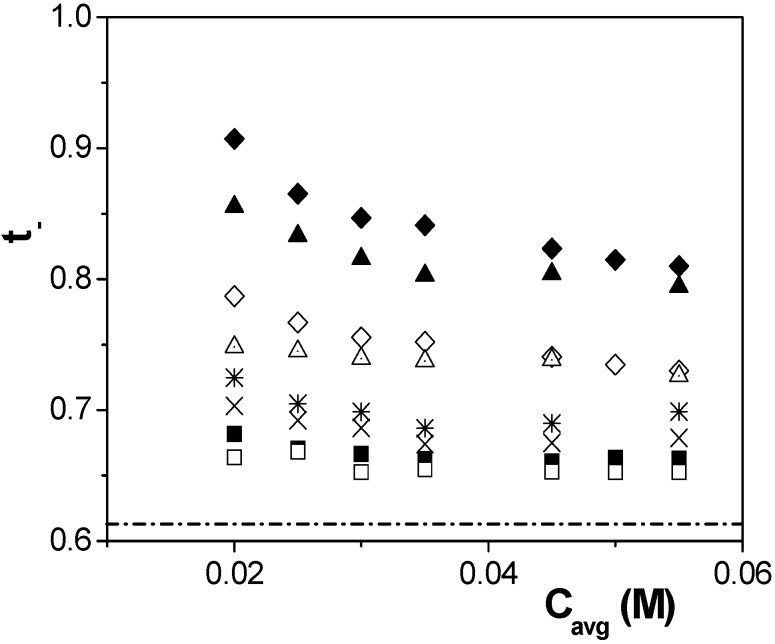
Anion transport number as a function of average solutions concentration determined by Equation (2) for stirred (dense symbols) and non-stirred (open symbols) solutions: Anopore (■, □); Al-Sf (♦, ◊); Al-Ox/Al_2_O_3_ (▲, ∆); and Al-Ox/SiO_2_ (_*_, ×).

Assuming that the membrane potential only corresponds to a diffusion potential associated to the different mobility of the ions into its porous structure, which is usually an adequate approximation for low charged membranes and high solution concentration [16], the value of the anion transport number *t*_−_ in the membrane for each pair of the measured solution concentrations (*C*_c_, *C*_v_) can be obtained by using Equation (2). Variation of t_-_ values with the average concentration (*C*_avg_ = (*C*_c_ + *C*_v_)/2) for the studied membranes under stirring and non-stirring solutions conditions is shown in Figure 7, where solution anion transport number (*t*_−_^0^) is also represented by a dashed-dot line.

According to these results, a reduction of around 12% in the value of the anion transport number through both alumina membranes, with low porosity/higher fixed charge, was obtained when measurements were performed without stirring the NaCl solutions, but its effect on the alumina membrane with similar pore size but higher porosity/lower fixed charge is only of 2% and practically independent of the concentration gradient. Moreover, the chemical modification of the membrane surface as a result of SiO_2_ coverage also decreases the concentration polarization effect, being the difference in transport number between stirred and non-stirred solution ~3%; this reduction seems to be directly related to differences in the NaCl/SiO_2_ electroaffinity when compared with the NaCl/Al_2_O_3_ interactions corresponding to the other membranes, even if similar pore size and porosity are considered.

## 3. Experimental Section

### 3.1. Membranes

Experimental NPAMs were synthesized by the two-step anodization process developed by Masuda *et al.* [20] and explained in detail elsewhere [21], starting from high purity Al foils (Al 99.999%) and employing a potentiostatic anodization method in acidic aqueous electrolites (0.3 M sulfuric acid at constant anodization voltage of 25 V for sample Al-Sf, while 0.3 M oxalic acid and anodization voltage of 40 V for sample Al-Ox). After this process, the remaining Al substrate was removed by wet chemical etching in a mixture of HCl and CuCl_2_, and the alumina barrier layer blocking the pores at the bottom was removed by reactive ion etching (RIE) in CF_4_/O_2_ plasma. Time duration of the second anodization step determines the thickness of the resulting NPAMs, which in the present work was adjusted to around 60 μm, approximately.

ALD coatings of the Al-Ox membranes were carried out in a BENEQ-TFS200 HPR reactor (Beneq, Vantaa, Finland), at 150 °C and operating in stop-mode (45 s exposure time, 60 s pump time) to ensure homogeneous coating along the membrane inner channels, using as precursors water (20 °C) and trimethylaluminium (20 °C) for Al_2_O_3_ deposition and 3-aminopropyltriethoxysilane (100 °C), water (20 °C) and ozone (20 °C) for SiO_2_ [22].

### 3.2. Surface Characterization by SEM

Tailor made alumina membranes were morphologically characterized by SEM micrographs of top and bottom surfaces together with the cross-section view carried out at 20 kV in a JEOL-6610LV (JEOL Ltd., Tokyo, Japan). The geometrical parameters of the NPAMs (pore size, porosity and spatial pore arrangement) were determined by using both ImageJ (National Institutes of Health, Bethesda, MD, USA) and WSxM (Nanotec Electronica S.L., Madrid, Spain) software for image analysis [23,24,25]. Samples were previously coated with a thin gold layer by means of a sputtering process to make them conductive.

### 3.3. Membrane Potential Measurements

Membrane potentials ∆Ф_mbr_), or equilibrium electrical potential difference between two NaCl solutions of different concentration (*C*_c_ and *C*_v_) at both membrane sides, were measured in a dead-end test cell similar to that described in [26], which basically consists of two glass half-cells with the membrane placed in the middle of both cells and two magnetic stirrers at the bottom of each cell to minimize the concentration-polarization effect at the membrane surfaces. An Ag/AgCl electrode (reversible to Cl^−^ ion) was placed in each half-cell and connected to a digital voltmeter (Yokogawa 7552, 1 GΩ input resistance, Tokyo, Japan), which allows the determination of the cell potential (∆*E*); these measurements were performed by keeping fixed the concentration of the solution at one side of the membrane (*C*_f_ = 0.01 M) and gradually changing the concentration of the solution at the other side (*C*_v_) from 0.01 M to 0.1 M, at room temperature (25 ± 2) °C, standard pH (5.8 ± 0.3) and solutions stirring rate of 540 rpm. Membranes were maintained overnight in contact with a 0.01 M NaCl solution to ensure pores filling, but it was renewed before starting the measurements. ∆Ф_mbr_ values were obtained by subtracting the electrode potential (∆Ф_elect_) to the measured cell potential values, that is, ∆Ф_mbr_ = ∆*E* − ∆Ф_elect_.

## 4. Conclusions

NPAMs with regular and well-defined pore radii (around 10 nm) but different porosity (between 5% and 30%) have been obtained by the two-step anodization method, and the diffusive transport across the membranes has been characterized by membrane potential measurements performed with NaCl solutions at different concentrations. These results show the influence of Donnan potential (co-ions exclusion) in the total value of membrane potential for the alumina samples, but this effect is clearly reduced with the increase of membrane porosity. The influence of electrolyte/membrane surface electroaffinity in the diffusive transport has also been demonstrated, taking into account the differences obtained for membranes with Al_2_O_3_ and SiO_2_ surfaces, but similar geometrical parameters. Moreover, differences in the membrane potential values obtained from measurements performed with stirred and non-stirred solutions and, consequently, in the values estimated for the anion transport numbers, clearly show the importance of hydrodynamic solution conditions in an adequate characterization of diffusive transport, which might appreciably affect the estimated parameters in the case of low pore radius and low porosity membranes.
